# Dr. Delavan E. Walker

**Published:** 1904-07

**Authors:** 


Dr. Delavan E. Walker, of Ilion, N. Y., a graduate of the University of Buffalo, 1882, died at his home after a prolonged illness, May 18, 1904, aged 49 years.

